# An Integrative Approach to Characterize the Early Phases of Dimethylhydrazine-Induced Colorectal Carcinogenesis in the Rat

**DOI:** 10.3390/biomedicines10020409

**Published:** 2022-02-09

**Authors:** Rita Silva-Reis, Catarina Castro-Ribeiro, Mariana Gonçalves, Tiago Ferreira, Maria João Pires, Carlos E. Iglesias-Aguirre, Adrián Cortés-Martín, María V. Selma, Juan Carlos Espín, Elisabete Nascimento-Gonçalves, Alexandra Moreira-Pais, Maria J. Neuparth, Francisco Peixoto, Eduardo Rosa, Adelina Gama, Rita Ferreira, Paula A. Oliveira, Ana I. Faustino-Rocha

**Affiliations:** 1 Centre for the Research and Technology of Agro-Environmental and Biological Sciences (CITAB), Inov4Agro, University of Trás-os-Montes and Alto Douro, 5000-801 Vila Real, Portugal; al69292@utad.eu (R.S.-R.); al61108@utad.eu (C.C.-R.); al59971@utad.eu (M.G.); al58092@utad.eu (T.F.); joaomp@utad.pt (M.J.P.); al34304@utad.eu (E.N.-G.); erosa@utad.pt (E.R.); pamo@utad.pt (P.A.O.); 2 Department of Veterinary Sciences, University of Trás-os-Montes and Alto Douro, 5000-801 Vila Real, Portugal; agama@utad.pt; 3 Laboratory Food & Health, CEBAS-CSIC, Campus de Espinardo, 30100 Murcia, Spain; ceiglesias@cebas.csic.es (C.E.I.-A.); acortes@cebas.csic.es (A.C.-M.); mvselma@cebas.csic.es (M.V.S.); jcespin@cebas.csic.es (J.C.E.); 4 LAQV-REQUIMTE, Department of Chemistry, University of Aveiro, 3810-193 Aveiro, Portugal; alexandrapais@ua.pt (A.M.-P.); ritaferreira@ua.pt (R.F.); 5 Research Center in Physical Activity, Health and Leisure (CIAFEL), Faculty of Sports, University of Porto, 4200-450 Porto, Portugal; mjoao.neuparth@ipsn.cespu.pt; 6 Chemistry Research Center, University of Trás-os-Montes and Alto Douro, 5000-801 Vila Real, Portugal; fpeixoto@utad.pt; 7 Associate Laboratory for Animal and Veterinary Science—AL4AnimalS, Animal and Veterinary Research Centre (CECAV), University of Trás-os-Montes and Alto Douro, 5000-801 Vila Real, Portugal; 8 Department of Zootechnics, School of Sciences and Technology, University of Évora, 7004-516 Évora, Portugal; 9 Comprehensive Health Research Center, 7004-516 Évora, Portugal

**Keywords:** gut microbiota, inflammation, oxidative stress

## Abstract

This study aimed to characterize an animal model of colorectal cancer (CRC) in the early stages of disease development. Twenty-nine male Wistar rats were divided into two control groups (CTRL1 and CTRL2), receiving EDTA–saline injections and two induced groups (CRC1 and CRC2), receiving 1,2-dimethylhydrazine (DMH) injections for seven consecutive weeks. CRC1 and CTRL1 were euthanized at the 11th week, while CRC2 and CTRL2 were euthanized at the 17th week. DMH treatment decreased microhematocrit values and IL-6, ghrelin, and myostatin serum levels. Histopathological analysis of intestinal sections showed that DMH-treated rats were characterized by moderate to severe epithelial dysplasia. An adenoma was observed in one animal (CRC2 group), and the presence of inflammatory infiltrate at the intestinal level was primarily observed in DMH-treated animals. DMH also induced Ki-67 immunoexpression. The gut microbiota analysis showed a higher abundance of Firmicutes, Clostridia, Clostridiales, Peptostreptococcaceae, *Blautia*, *Romboutsia,* and *Clostridium sensu stricto* in CRC than CTRL rats, whereas Prevotellaceae, *Prevotella*, *Akkermansia,* and *Lactobacillus* levels were more prevalent in CTRL animals. Our results suggest that this model could be helpful to investigate chemoprevention in the early stages of CRC.

## 1. Introduction

CRC is the third most common cancer and the second leading cause of cancer death worldwide, with an estimated 935,173 deaths in 2020 [1]. Most CRC cases are caused by random mutations, whereas others are caused by known genetic alterations [2]. The CRC high mortality rate can be linked to a lack of understanding of the potential of animal models available for its research and, consequently, their underuse [3].

The available animal models of CRC have been used for more than eight decades to better understand the molecular events involved and evaluate the efficacy of many treatments [4]. Despite this, most of the research in these models has been limited to histological characterization [5,6,7], few studies on oxidative stress [8,9] and gut microbiome analyses [10], and even fewer regarding the early phases of the tumorigenic events. In view of this, an integrative and complementary approach is needed, which will provide a better characterization and understanding of the model, with potentially more conclusive benefits for CRC patients [11,12]. 

This research aims to provide a thorough characterization of the animal model of CRC induced by DMH in the early phases of disease development, making it more accurate, objective, and valuable for future studies.

## 2. Materials and Methods

### 2.1. Animals and Chemicals

Twenty-nine male Wistar rats, weighing 200–250 g and aged seven weeks, were acquired from Charles River (France). Sigma-Aldrich^®^ (Saint Louis, MO, USA) provided DMH, which was prepared immediately before its use in a saline solution containing 0.9% of NaCl (B. Braun, Melsungen, Germany) and 1 mM of ethylenediaminetetraacetic acid (EDTA; E5134, Sigma-Aldrich^®^, Saint Louis, Missouri, USA) [10]. During the study, the rats were kept in controlled conditions of temperature (20 ± 2 °C), relative humidity (50 ± 10%), and light-dark cycle (12:12-h). The rats were housed in groups of three to five animals *per* cage (1500UEurostandard Type IV S cages, Tecniplast, Buguggiate, Italy). Food (Diet Standard 4RF21^®^, Mucedola, Italy) and tap water were provided ad libitum throughout the study. Corncob was used as bedding and was changed weekly. Polyvinyl chloride tubes were placed into the cages as environmental enrichment for the animals. The viruses and bacteria specified in the Supplementary Materials (Table S1), as well as endo and ectoparasites, were not found in the rats. 

### 2.2. Experimental Groups and Treatments

**Figure 1:**
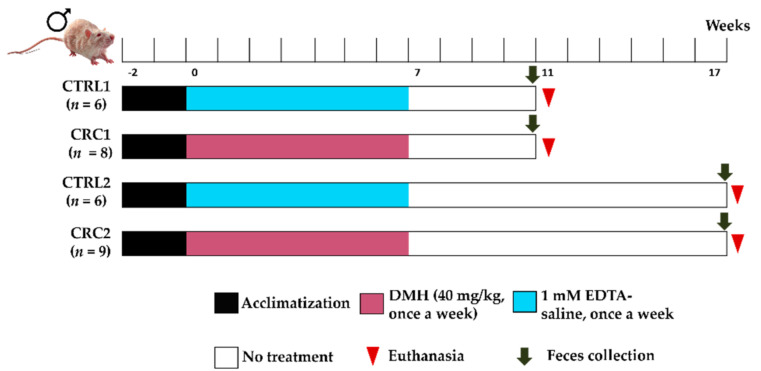
The experimental protocol implemented for the study of DMH-induced CRC.

After two weeks of acclimatization to the lab conditions, animals were randomly assigned to four groups: Control (CTRL) 1 (*n* = 6), CRC1 (*n* = 8), CTRL2 (*n* = 6), and CRC2 (*n* = 9). This protocol was adapted from Zhu et al. [10]. Once a week, for seven weeks, animals from both groups CRC received an intraperitoneal injection of DMH [13], at a dose of 40 mg/kg of body weight (BW) [14], prepared immediately before its use as described above. Animals from CTRL groups were intraperitoneally injected with the vehicle (1 mM EDTA-saline) (Figure 1). A larger number of animals was included in CRC groups because of the higher probability of death due to the DMH administration. Individual animal weights, as well as food and water consumption, were registered once a week using a top-loading scale (Mettler^®^ Toledo PM4000, Midland, Canada). The study was previously approved by an Ethics Review Body (“ORBEA-Orgão Responsável pelo Bem-Estar e Ética Animal”, reference: 142-e-CITAB-2017/2017-09-25”) and the Portuguese Competent Authority (“DGAV-Direção Geral de Alimentação e Veterinária”, approval number 010535, 1 June 2018). The work was performed following Portuguese law (Decree-Law No. 113/2013) on the protection of animals used for scientific purposes. 

### 2.3. Samples Collection and Necropsy

On the day before euthanasia, the animals were placed in metabolic cages to collect stool samples. The feces were stored at −80 °C to study the gut microbiota. Animals from CRC1 and CTRL1 groups and those from the CRC2 and CTRL2 groups were euthanized eleven and seventeen weeks after the first DMH or EDTA-saline treatment, respectively (Figure 1). Following the Federation of European Laboratory Animal Science Associations (FELASA) recommendations, all animals were sacrificed by an intraperitoneal overdose of pentobarbital sodium (Eutasil, CEVA, Libourne, France), followed by exsanguination by cardiac puncture. A complete necropsy was performed on each animal. Blood samples were collected and placed in heparinized capillary tubes (Deltalab, Barcelona, Spain) for microhematocrit determination and in anticoagulant-free tubes (Deltalab, Barcelona, Spain) to obtain serum. The serum tubes were centrifuged at 3000 rpm (HeraeusTM LabofugeTM 400R, Thermo Scientific, Waltham, MA, USA) for 15 min under refrigeration (4 °C) and stored at −80 °C for biochemical analysis. All organs were collected and weighed. The intestine portions were individualized and weighed separately. The colon was opened longitudinally, carefully cleaned with saline to eliminate any leftover intestinal content before being fixed flat in 10% buffered formalin. The remaining organs were also immersed in buffered formalin for at least 24 h. 

### 2.4. Microhematocrit Analysis

The capillary tubes were centrifuged in a micro-hematocrit centrifuge (PrO-Vet^®^, Centurion Scientific, Chichester, UK) for 5 min at 1200 rpm, and then the packed red cell volume was measured using a ruler. 

### 2.5. Serum Biochemical Analysis and Immunoblotting

Serum albumin, total protein, cholesterol, glucose, and alanine aminotransferase (ALT) were quantified in an automated auto-analyzer (Prestige 24i, Cornay PZ). The circulating levels of C-reactive protein (CRP), interleukin-6 (IL-6), myostatin, and ghrelin were determined by immunoblot. In brief, serum samples were diluted (1:30) in tris-buffered saline (TBS). After activating the nitrocellulose membranes in 10% (*v*/*v*) methanol, 50 μL of each sample was transferred to the nitrocellulose membrane (0.45 μm porosity, Amersham™ Protran™, GE Healthcare, Darmstadt, Germany) using a Slot-blot system under vacuum. The membrane was then incubated for one hour in blocking solution (5% (*w*/*v*) nonfat dry milk in TBS with 0.5% Tween 20 (TTBS)) and was incubated with the primary antibody diluted 1:1000 in 5% (*w*/*v*) nonfat dry milk in TTBS for one hour. The primary antibodies used were rabbit polyclonal anti-GDF8/myostatin (ab98337, Abcam, Cambridge, UK), rabbit polyclonal anti-ghrelin (ab64325, Abcam, Cambridge, UK), rabbit polyclonal anti-CRP (ab65842, Abcam, Cambridge, UK), and rabbit polyclonal anti-IL-6 (ab6672, Abcam, Cambridge, UK). After three washes (10 min each) of the membrane with TTBS, it was incubated with a secondary antibody (anti-rabbit; GE Healthcare, Chicago, IL, USA) for one hour and washed again with TTBS three times. Membranes were exposed to ECL solution (WesternBright ECL; Advansta, CA, USA) for two minutes. The images were obtained by chemiluminescence in a ChemiDoc XR+ system (Bio-Rad, Advansta, CA, USA).

### 2.6. Histopathological Analysis

After fixation, collected organs were processed for routine histopathological analysis. Formalin-fixed intestinal samples from both groups CRC were previously stained with 0.2% methylene blue for 5 min for preneoplastic aberrant crypt foci (ACF) screening and quantification under a stereomicroscope (Leica Zoom 2000, Wetzlar, Germany) [15]. A 4-μm section of paraffin-embedded of each sample was stained with hematoxylin and eosin (H&E) and observed blindly under a light microscope by a veterinary pathologist, considering the criteria proposed by Nolte et al. [16]. The degree of inflammation of small intestine and cecum was evaluated on a scale from 0 to 3 (0 = no inflammation, 1 = mild, 2 = moderate, and 3 = severe). The degree of inflammation of the colon was evaluated according to Tian et al. [17]. Briefly, the severity of inflammation was assessed on a scale from 0 to 3 (0 = no inflammation, 1 = mild, 2 = moderate, and 3 = severe) and the thickness of inflammatory involvement was scored from 0 to 3 (0 = no inflammation, 1 = mucosa, 2 = mucosa plus submucosa, and 3 = transmural). Furthermore, the severity of epithelial damage was scored from 0 to 3 (0 = intact epithelium, 1 = disruption of architectural structure, 2 = erosion, and 3 = ulceration) and extent of lesions was also scored from 0 to 3 (0 = no lesions, 1 = punctuate, 2 = multifocal, and 3 = diffuse). 

### 2.7. Ki-67 Immunostaining

The colon was stained with Ki-67 to assess cell proliferation. The immunohistochemical detection of Ki-67 was performed using the standard protocol of the NovoLink^TM^ Polymer Detection System (RE7150-K, Leica Biosystems, Newcastle, UK). Briefly, 4-μm paraffin-embedded colon sections were deparaffinized in xylene, hydrated in decreasing ethanol solutions, and rinsed in distilled water. Antigen retrieval was performed by heating the slides in a 0.01 mol/L citrate buffer (pH 6.0) in the microwave (3 cycles of 5 min, 750 W, with stirring) and then washed with phosphate-buffered saline (PBS). The endogenous peroxidase was inactivated with hydrogen peroxide 3% for 30 min, followed by protein blocking (0.4% casein in serum with phosphate buffer) for five minutes. Samples were then incubated overnight at 4 °C with the anti-rabbit primary antibody anti-Ki67 (ab16667, Abcam, UK) at 1:200 dilution. After PBS washing, slides were incubated with Post Primary Block to activate the polymer penetration for 30 min. For the detection step, the slides were incubated with the NovoLink Polymer for 30 min, washed with PBS, and then exposed to DAB chromogen for 10 min. After washing in running water for about ten minutes, the slides were counterstained with Gill’s hematoxylin (0.02%) for one minute. Finally, dehydration was performed with ethanol solutions of increasing concentrations, diaphanization with xylol, and mounted with Entellan^®^. The QuPath program (Quantitative Pathology & Bioimage Analysis, v0.1.2 for Windows, Queen’s University of Belfast, Belfast, Northern Ireland) was used to calculate the percentage of Ki-67 immunostaining.

### 2.8. Antioxidant Enzymes Activity

A portion of the colon was used to analyze oxidative stress markers, specifically superoxide dismutase (SOD) and catalase (CAT). After thawing the sample, the mucosa was separated from the rest of the colon by scraping with the back of a scalpel, weighed, and diluted in cold Tris-HCl buffer (10 mM, pH 7.4). Subsequently, the mixture solution was homogenized with an ultrasonic processor (3 × 15 s, intermittent 15 s) in an ice bath, and post-mitochondrial supernatant was obtained after centrifugation at 8000 rpm, 4 °C, for 10 min (Sigma Laborzentrifugen™ 2-16K centrifuge, Osterode am Harz, Germany). Next, the supernatant was transferred into another tube and again centrifuged at 12,000 rpm, 4 °C, for 10 min (Sigma Laborzentrifugen™ 2-16K centrifuge, Osterode am Harz, Germany). Finally, the supernatants were used to analyze the enzymatic activity of SOD and CAT.

SOD activity (EC 1.15.1.1) was assessed with minor modifications according to Payá et al. (1992) [18], based on its ability to inhibit the photochemical reduction of a chromophore, nitroblue tetrazolium chloride (NBT). This is possible because the NBT allows the detection of superoxide ion production, which occurs through the oxidation of hypoxanthine to xanthine and later to uric acid through the action of xanthine oxidase. The reaction mixture (1 mL) consisted of phosphate buffer (50 mM KH_2_PO_4_, 1 mM EDTA pH 7.4), 10 μL of hypoxanthine (10 mM), and 10 μL of NBT (10 mM). The reaction was initiated by adding xanthine oxidase (10 % (*v*/*v*)) to enzymatic extract at 30 °C. The enzymatic kinetics was monitored spectrophotometrically (Varian Cary^®^ 50 UV-Vis Spectrophotometer) at 560 nm for 1 min to obtain the blank value. Subsequently, 40 μL of the sample was added to the mixture, and the reaction was monitored under the same conditions as previously mentioned. The results were expressed in U/min/mg/protein, where one unit of SOD activity (1 U) is defined as the amount of SOD that inhibits 50% of the reduction of NBT to formazan.

CAT activity (EC 1.11.1.6) was assayed using a modified version of the method described by del Río et al. [19]. First, the enzymatic activity was evaluated on a Clark type oxygen electrode that allowed the observation of O_2_ production, resulting from the degradation of hydrogen peroxide (H_2_O_2_). The reaction mixture consisted of a phosphate buffer (50 mM KH_2_PO_4_, pH 7.0) and 10 μL of H_2_O_2_ (3 % *w*/*v*). The mixture was incubated in the chamber at 30 °C for about 1 min. After this time, 5 μL of the sample was added so that the final volume was 1 mL. The reaction was monitored for about two minutes. The results were expressed in mmol H_2_O_2_ consumed min^−1^ mg protein^−1^.

The results of the enzymatic activities were expressed as a function of the protein content of the samples, following its determination applying the Bradford method and using bovine serum albumin (BSA) as standard [20].

### 2.9. Gut Microbiota Analysis

Bacterial DNA from rat stool samples was isolated using the NucleoSpin™ Tissue DNA Purification Kit (Macherey-Nagel, Düren, Germany). Rat gut microbiota analysis was carried out after sequencing gene-specific sequences V3-V4 variable region of 16 rRNA gene. Metagenomic sequencing was performed following Illumina protocols (Illumina Inc., San Diego, CA, USA) with a read length of 2 × 300 bp paired-end run (MiSeq Reagent Kit v3, Illumina Inc.) on a MiSeq-Illumina platform (FISABIO sequencing service, Valencia, Spain). Data processing (including quality assessment, removal of chimeric or misaligned sequences, and the alignment and clustering of sequences) to obtain alpha-diversity and taxonomic classification with 97% identity from phylum to genus were carried out as described previously [21]. Briefly, quality assessment was performed using a prinseq-lite program [22] and applying the following parameters: min_length: 50; trim_qual_right: 30; trim_qual_type: mean; trim_qual_window: 20. R1 and R2 from Illumina sequencing were joined using fastq-join from the ea-tools suite. The data obtained in the fastaq format were processed using the Galaxy tool [23]. Chimeric sequences and sequences that could not be aligned were also removed from the data set and filtered out with UCHIME [24]. The clustered sequences were utilized to construct Operational Taxonomic Units (OTUs) tables with 97 % identity, and representative sequences were classified into the respective taxonomical level from phylum to genus using the RDP classifier [25].

Analyses with RDPipeline (http://pyro.cme.msu.edu/ accessed on 11 December 2020) involved 16S rRNA gene sequence alignment (Aligner), 16S rRNA gene sequence clustering (Complete Linkage Clustering), and alpha-diversity indexes (Shannon Index and Chao1 estimator) at the genus level. Alpha-diversity indexes (Chao1 and Shannon), based on a randomly selected 87,349 reads per sample, were used to estimate the samples’ richness and diversity at the genus, family, and phylum levels. Shannon and Chao 1 indexes at phylum and family levels were calculated by PAST version 2.17c (http://folk.uio/ohammer/past accessed on 11 December 2020). The LDA Effect Size (LEfSe) algorithm with the online interface Galaxy (http://huttenhower.sph.harvard.edu/galaxy/root accessed on 11 December 2020) was used to identify taxa with differentiating abundance among groups. LEfSe identified statistically different features among the different groups and performed non-parametric factorial Kruskal–Wallis sum-rank tests and Linear Discriminant Analysis (LDA) to determine whether these features were consistent concerning the expected behavior of the different treatments.

### 2.10. Statistical Analysis

The relative weight of the intestine was calculated using the following formula: (organ weight)/(rat’s final body weight–intestinal content weight). GraphPad Prism^®^ software for Windows (version 8.0.1, San Diego, CA, USA) was used to analyze animals’ body weight, food and water consumption, organs’ weight, microhematocrit, serum markers, intestine inflammation, Ki-67 immunoexpression, and antioxidant enzymes activity. The differences among groups were determined using an analysis of variance (ANOVA) followed by a Bonferroni post hoc. These data are presented as mean ± standard deviation (SD). The differences were considered significant at *p* < 0.05. 

Linear Discriminant Analysis (LDA) Effect Size (LEfSe) was carried out using the online interface Galaxy http://huttenhower.sph.harvard.edu/galaxy/root accessed on 11 December 2020 to determine specific differences in relative abundances in microbial taxa between two groups (CTRL1 vs. CRC1, CTRL2 vs. CRC2, etc.). Principal component analysis (PCA) was performed with SPSS software v.26.0 (SPSS. Inc, Chicago, IL, USA) using discriminant microbial groups (phylum, family, and genus) obtained in the LEfSe analysis. ANOVA followed by Dunnett T3 *post hoc* was used to find differences in principal components (PCA1 and PCA2) between groups (CTRL and CRC).

## 3. Results

### 3.1. Characterization of Animals’ Systemic Adaptations to CRC Induction

Five animals from the DMH-induced CRC groups (two from the CRC1 group (10th and 14th week of the study) and three from the CRC2 group (14th, 16th, and 17th week of the study)) died of hemorrhagic enteritis during the experiment. Data from these animals were not included in the study. There were no significant variations in body weight, food, or water intake among groups (*p* > 0.05). However, the relative weight of the small intestine was significantly higher in both CRC groups induced when compared to CTRL groups (*p* < 0.0001; Table 1). Besides, the cecum relative weight of the CRC2 group was considerably higher compared with CTRL groups (*p* < 0.01). No significant differences were observed in the relative weight of the remaining organs among groups (*p* > 0.05; data not shown).

Animals from the CRC1 group exhibited a higher microhematocrit value when compared with those also exposed to DMH and sacrificed later (CRC2 group; *p* < 0.01, Table 2). 

Serum levels of albumin, total protein, cholesterol, glucose, and ALT were similar among groups (*p* > 0.05). The highest IL-6 serum levels were observed in the CTRL2 group (*p* < 0.05). Although the differences did not reach the level of statistical significance, CRP levels were higher in both CTRL2 and CRC2 groups when compared with the CTRL1 and CRC1 groups (*p* > 0.05). Like CRP, the myostatin levels were higher in both CTRL2 and CRC2 groups when compared with the CTRL1 and CRC1 groups. Ghrelin levels were higher in the CTRL2 groups when compared with those from CTRL1 and CRC1 groups (*p* < 0.05 and *p* < 0.01, respectively; Table 3).

### 3.2. DMH Induced Preneoplastic Lesions Associated with CRC

**Figure 2:**
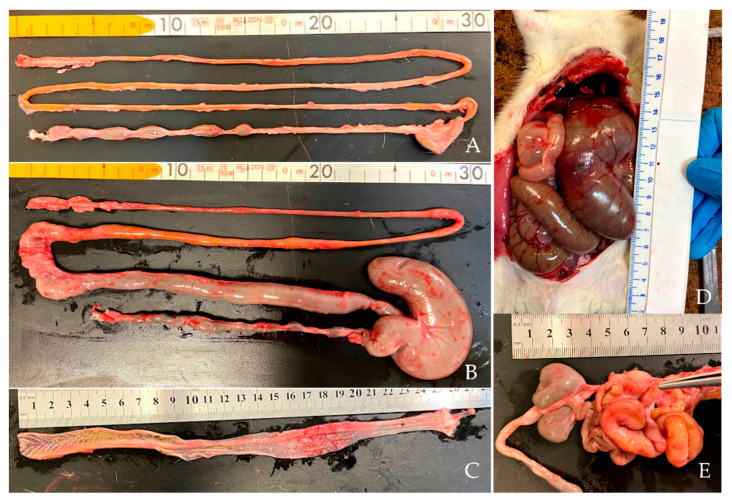
Macroscopic appearance of small and large intestines. (**A**) Intestine without lesions (CTRL1 group). (**B**) Hemorrhagic distension of small intestine and cecum (CRC2 group). (**C**) Colon + rectum with inflammation in the intestinal lumen (CRC2 group). (**D**) Dilated cecum showing a focal necrotic lesion before opening (CRC2 group). (**E**) Mesenteric lymph node enlargement (arrow), and small intestine and cecum distension (CRC2 group).

Control groups had no macroscopic lesions, whereas several animals from both induced groups had abdominal distension, which was associated with intestine segmental distension, affecting the small intestine (4/6 and 6/6 animals from CRC1 and CRC2 groups, respectively) and cecum (3/6 and 4/6 animals from CRC1 and CRC2 groups, respectively). In addition, two focal hemorrhagic/necrotic lesions were observed in the cecum of animals from CRC1 (*n* = 1) and CRC2 (*n* = 1) groups (Figure 2). No macroscopic tumors were observed in the rat colons, either from control or DMH-treated groups; one rat from the CRC2 group exhibited a hemorrhagic focal lesion. 

**Figure 3:**
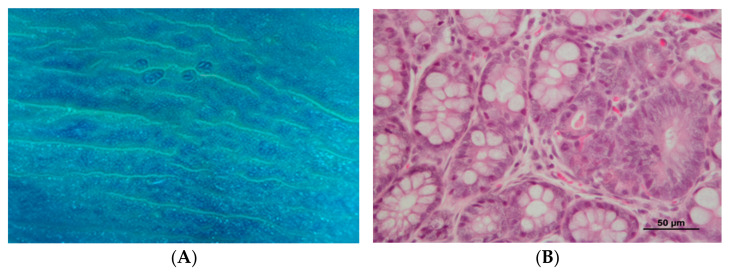
Colon analysis. (**A**) Presence of aberrant crypt foci (ACF) in the colon of CRC2 group (methylene blue staining, stereomicroscope); (**B**) Colon with the presence of focal mild epithelial dysplasia, CRC1 group (HE staining).

Colon ACF were easily visualized by staining with 0.2% methylene blue, slightly elevated above the surrounding mucosa, and usually demonstrating characteristic oval or slit-like orifices (Figure 3A). The average number of ACF was 24.00 ± 4.06 *per* animal in the CRC1 group and 24.83 ± 5.33 in the CRC2 group (*p* > 0.05).

A moderate to marked mononuclear inflammatory infiltrate was frequently observed in the small intestine and cecum of both DMH-treated groups (CRC1 and CRC2), often associated with epithelial hyperplasia or villous atrophy, and fusion in the small intestine (Table 4). Besides, small intestine enteritis was observed in two animals from the CRC1 group and one animal from the CRC2 group, and typhlitis was diagnosed in one animal from the CRC1 group and two animals from the CRC2 group.

No histopathological lesions were observed in the colon of animals from control groups. Preneoplastic lesions were observed in both DMH-treated groups, with neoplastic proliferation only present in the long-term experiment (CRC2 group). Half of the animals belonging to the CRC1 group (3/6) presented mild to moderate dysplasia foci. The incidence of neoplasia (adenoma) was 16.7% (1/6) in the CRC2 group, the only group with this lesion. In the same group, one animal also exhibited severe dysplasia (16.7%), and two presented mild to moderate dysplasia foci (33.3%) (Table 5, Figure 3B).

The control groups (CTRL1 and CTRL2) were injected with EDTA–saline solutions, and the CRC groups (CRC1 and CRC2) were injected with DMH. Groups 1 and 2 were euthanized 11 and 17 weeks after the first administration, respectively.

**Figure 4:**
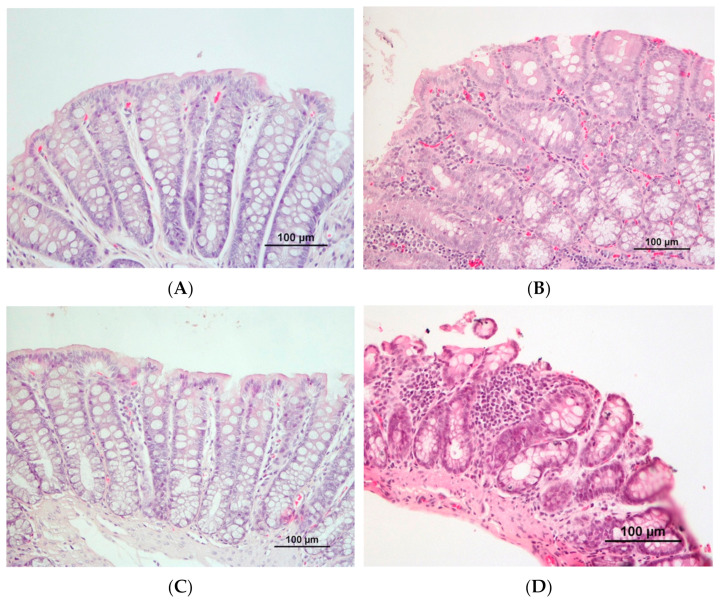
Evaluation of colon inflammation. Colon from controls ((**A**), CTRL1, and (**C**), CTRL2) exhibiting a mild inflammatory infiltrate, affecting the mucosa, with no disrupted architectural structure. Colon from CRC animals ((**B**), CRC1, and (**D**), CRC2), showing moderate mononuclear inflammatory infiltrate, especially affecting the mucosa, with disruption of architectural structure and erosion (HE staining).

Most animals presented some degree of inflammation in the colon (Table 6, Figure 4). The colon of animals from the CRC groups presented a mild to moderate inflammatory infiltrate, frequently affecting the submucosa, with disruption of architectural structure and occasional erosion. In contrast, controls usually exhibited a mild inflammatory infiltrate, affecting the mucosa, with rare disrupted architectural structure. In the CRC groups, the inflammatory lesions were mainly multifocal and occasionally diffuse, while in the respective controls, they were punctuated and rarely with a multifocal pattern.

**Figure 5:**
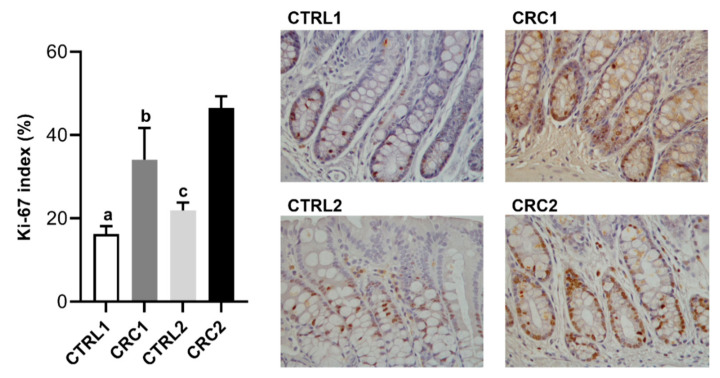
Ki-67 immunoexpression in rat’s colon. At left, Ki-67 index (percentage) observed in the CTRL and CRC groups. Data are presented as mean ± SD. ^a^ Statistically different from CRC1 group (*p* < 0.001); Statistically different from CRC2 group (*p* < 0.001); ^b^ Statistically different from CTRL2 group (*p* < 0.01); Statistically different from CRC2 group (*p* < 0.01); ^c^ Statistically different from CRC2 group (*p* < 0.001). On the right, representative histological images of Ki-67 immunolabelling of rat’s colon, with Ki-67 labeled epithelial cells showing brown nuclear positivity (Gill’s hematoxylin, 400×). The control groups (CTRL1 and CTRL2) were injected with EDTA–saline solutions and the CRC groups (CRC1 and CRC2) were injected with DMH. Groups 1 and 2 were euthanized 11 and 17 weeks after the first administration, respectively.

Regarding the expression of the cell proliferation marker Ki-67 in intestine sections, CRC1 and CRC2 groups had a significantly higher Ki-67 immunolabelling when compared with control groups (*p* < 0.001, Figure 5).

### 3.3. Effect of DMH on the Activity of Colonic Antioxidant Enzymes

Although the differences between control and CRC groups did not reach statistical significance (*p* > 0.05), the treatment with DMH decreased the antioxidant levels of the enzymes SOD and CAT (Table 7).

No statistically significant differences were found (*p* > 0.05). The control groups (CTRL1 and CTRL2) were injected with EDTA–saline solutions and the CRC groups (CRC1 and CRC2) were injected with DMH. Groups 1 and 2 were euthanized 11 and 17 weeks after the first administration, respectively.

### 3.4. Impact of DMH-Induced CRC on the Rat Gut Microbiota

We explored differences in the gut microbiota among the different rat groups. We set a threshold on the logarithmic LDA score for discriminant features at 3.6 to perform a more restrictive analysis focused on the distinctive microbial groups with greater abundance differences between groups. 

**Figure 6:**
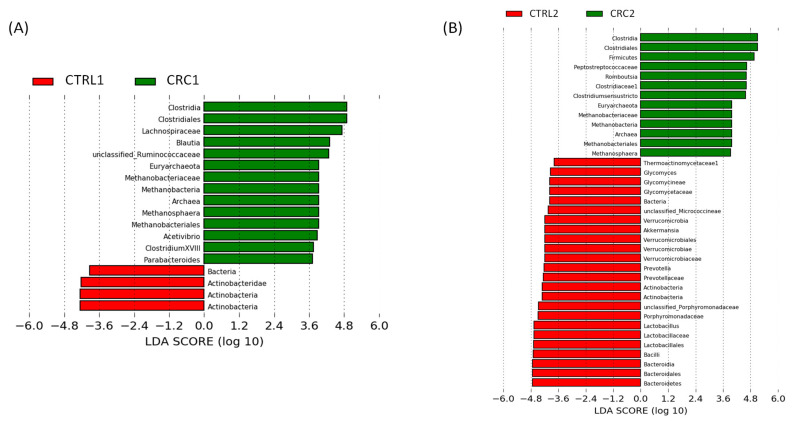
Linear discriminant analysis (LDA) effect size (LEfSe) of gut microbiota showing significant differences in the fecal microbiota of control (CTRL) and DHMH-induced colorectal cancer (CRC) rats. (**A**) Rats at 20 weeks of age and (**B**) rats at 26 weeks of age. Red bars, CTRL groups; Green bars, CRC groups.

Firstly, we compared CTRL1 vs. CTRL2 groups and CRC1 vs. CRC2 groups. The CTRL2 group showed higher Proteobacteria phylum levels than CTRL1 (Figure 6A), while the Bacteroidetes phylum (Bacteroidia class, Bacteroidales order) and the *Clostridium* cluster XVIII were more abundant in CRC1 than in CRC2 (Figure 6B). 

Then, we compared groups CTRL vs. CRC. Rats from the CRC1 group showed higher levels than CTRL1 of the Archaea domain, and the genera (i) *Blautia* (including also differences at higher taxonomic levels: Lachnospiraceae family, Eubacteriales order and Clostridia class), (ii) *Methanosphaera* (including also differences at higher taxonomic levels: Methanobacteriaceae family, Methanobacteriales order, Methanobacteria class, Euryarchaeota phylum, Archaea domain), (iii) *Acetivibrio*, (iv) *Parabacteroides* and (v) *Clostridium* cluster XVIII. On the contrary, the Bacteria domain and the Actinobacteria phylum were more abundant in CTRL1 vs. CRC1 (Figure 6A). Therefore, the gut microbiota’s composition was altered by DMH administration. 

Similar to the differences between CRC1 vs. CTRL1, an increased abundance was observed in CRC2 vs. CTRL2 for Clostridiales order and Clostridia class, and differences at the family level (Clostridiaceae) and phylum level (Firmicutes) were observed. Furthermore, other genera of the Clostridiaceae family, such as *Romboutsia* and *Clostridium sensu stricto*, were also increased in CRC2 vs. CTRL2. Like the differences between CRC1 vs. CTRL1, an increased abundance was observed in CRC2 vs. CTRL2 for *Methanosphaera* (including also differences at higher taxonomic levels: its Methanobacteriaceae family, Methanobacteriales order, Methanobacteria class, Euryarchaeota phylum, Archaea domain). An increased abundance was also observed in CTRL1 vs. CRC1 for the Bacteria domain and the Actinobacteria phylum. However, CTRL2 also showed higher levels than CRC2 in other bacterial groups: the genera *Glycomyces* (Glycomycetaceae family), *Akkermansia* (Verrucomicrobiaceae family), *Prevotella* (Bacteroidetes phylum, Prevotellaceae family), and *Lactobacillus* (Lactobacillaceae family), as well as the Porphyromonadaceae family (Figure 6B). Overall, the abundance of these latter bacterial groups decreased in CRC2 vs. CTRL2, which was the main reason for finding more differences in CTRL2 than in comparing CTRL1 vs. CRC1.

**Figure 7:**
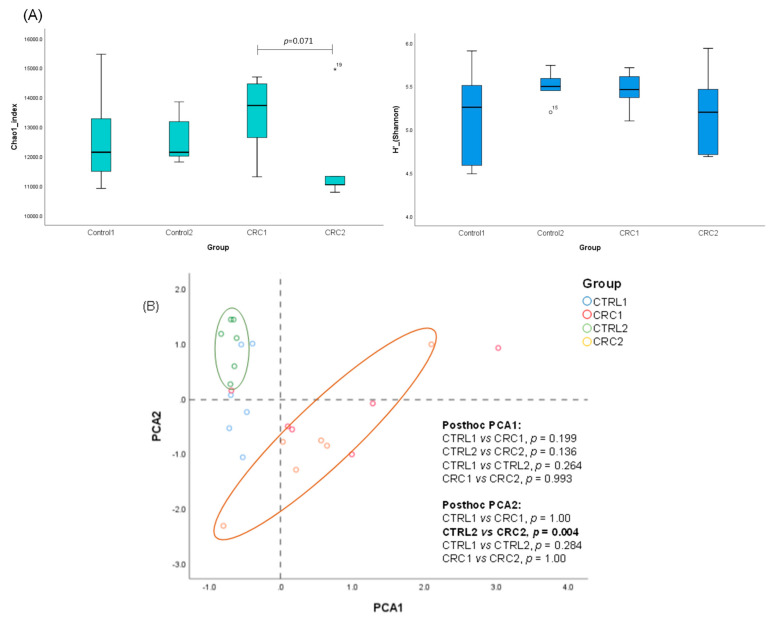
(**A**) Alpha diversity comparisons of fecal microbiomes collected from the different CTRL and CRC groups. (**B**) PCA and clustering analysis showing differences between CTRL and CRC groups.

The alpha-diversity (Shannon and Chao1 indexes) was evaluated for each group at each timepoint, and only the Chao1 index showed a marginally significant difference when CRC1 and CRC2 rats were compared (*p* = 0.071) (Figure 7A). Next, we performed a PCA with the main differential microbial groups (phylum, family, and genus) obtained in the LEfSe analysis (LDA score > 2) to check a possible microbial clustering upon treatments, i.e., EDTA-saline vs. DMH (Figure 7B). Only the PCA2 showed a statistically significant difference for CTRL2 vs. CRC2 (*p =* 0.004), mainly driven by the Bacteroidetes phylum, Prevotellaceae family, and the *Prevotella* and *Akkermansia* genera, among others, in CTRL2, and the Firmicutes phylum, Clostridiaceae family, and the *Clostridium sensu stricto* and *Anaerobacter* genera, among others, in CRC2.

## 4. Discussion

Chemical-induced CRC models have been successfully used to track the progression of CRC in Wistar rats [10,26]. This research intended to analyze an old model by using new tools. Considering current knowledge, we aim to have an animal model of chemical induction of colorectal cancer fully characterized through an integrated approach. The protocol of the present study was based on that previously performed by Zhu et al. [10], assessing the CRC progression over time. Although cancer research on animals is considered a procedure that causes great discomfort and stress, we did not anticipate such a high number of deaths (29% of mortality). The animals’ mean body weight, as well as their food and water intake, were not significantly different among groups. Despite being a frequent metric used to determine the severity of cancer in animal models, the animals’ body weight has some limitations because it can be hidden by the development of ascites or an increase in tumor mass [27]. Still, no significant changes were observed in serum ghrelin and myostatin levels of CRC animals, markers of appetite, and muscle catabolism, respectively, which support no alterations in body weight. Differences were expected at advanced stages of CRC, as previously reported [28,29]. 

The lower levels of hematocrit observed in the CRC2 group (Table 2) agree with Jrah Harzallah et al. [30]. These low levels can suggest anemia [31] due to several factors, namely blood loss in stools, inflammation in the small intestine, cecum, or colon, as was histologically identified, or systemic inflammation. However, the analysis of serum CRP, albumin, and IL-6, inflammatory markers linked to CRC progression and survival [32], did not support systemic inflammation at these stages of the disease. Previous findings showed higher CRP and IL-6 serum levels in DHM-induced groups than controls but in more advanced stages of the disease [33,34]. In fact, although there was no evidence of systemic inflammation, an increase in local inflammation (evaluated according to Tian et al. [17] was noted in the cecum, small intestine, and colon of animals belonging to the groups induced by DMH when compared to the respective controls. 

Macroscopic and microscopic alterations were noticed in the intestine. Indeed, DMH administration induced the development of multiple ACF in the colonic mucosa of all DMH-treated rats. ACFs are the first lesions detected microscopically in CRC carcinogenesis, commonly used as biomarkers during the early stages of the disease [35]. Our results are supported by previous studies showing an ACF increase in response to DMH [15,36]. The histological evaluation of ACF corresponded to dysplastic lesions. This may be because ACF comprise heterogeneous histological lesions, including non-dysplastic and dysplastic ACF, being the latter considered as true preneoplastic lesions, which more frequently harbor gene mutations [31]. Only one animal (from the CRC2 group) presented a neoplastic lesion at the 17th week, classified as a benign proliferation (adenoma). Inversely, Zhu et al. [10] found the first adenoma at the 12th week and described the presence of adenocarcinomas at the 20th week. These differences can be due to the different ages of animals used in the protocols, i.e., the animals used by Zhu et al. were four weeks old, and the animals used in our study were seven weeks of age. A study performed by Jia and Han [37] presented a 100% incidence of tumors at the end of 32 weeks, using a lower dose when compared to our study (20 mg/kg vs. 40 mg/kg). However, like our investigation, other studies with lower carcinogen doses and short duration only reported preneoplastic lesions [34,38]. The comparative analysis of these studies suggests that our protocol can be improved by reducing the dose of carcinogen administered, opting for subcutaneous injections to facilitate the weekly procedure, choosing younger animals, and having a longer protocol. CRC carcinogenesis occurs in multiple phases, characterized by the presence of uncontrolled crypt cell proliferation [39]. The nuclear protein Ki-67 has become widely used in routine CRC histopathological research as a marker, strongly associated with tumor cell proliferation and growth [40]. Our results revealed that DMH induced a higher proliferation of epithelial cells in the colon of the animals at both euthanasia timepoints and are supported by previous studies that also used DMH to induce colorectal carcinogenesis [15,41,42]. 

Furthermore, in the CRC groups, the relative weight of the colon, small intestine, and cecum was higher than in the controls. This occurrence could be linked to the presence of a mononuclear inflammatory infiltrate. According to our findings, DMH produced lesions in various organs, including the small intestine and cecum. These lesions were characterized by an increase in the inflammatory infiltrate, which was sometimes associated with hemorrhage or necrosis. Despite being less common than lesions in the colon, dysplastic and hyperplasic lesions in the small intestine have been found in earlier investigations [43,44,45]. The inflammation observed in these organs may have been initiated by pathogens characteristic of enteritis [46]. Chronic inflammation is a known risk factor for CRC. Inflammatory cells and associated mediators (e.g., IL-6 and ROS) form a microenvironment that may enhance DNA damage in epithelial cells [47]. The exact mechanism that links chronic inflammation to CRC carcinogenesis is not entirely understood but seems to involve cyclooxygenase-2 (COX-2) and nuclear factor kappaB (NF-κB) [48]. Still, the local effects of chronic inflammation seem to target the intestinal microbiota and induce changes in microbes’ expansion [47]. 

DMH is metabolized in the liver resulting in its metabolites AOM and methylazoxymethanol. These are then transported to the colon, where the final metabolite, diazonium, can methylate DNA bases, generating hydroxyl radicals (•OH) or hydrogen peroxide (H_2_O_2_) [9]. SOD and CAT are two of the main enzymes belonging to the cells’ antioxidant system and play a key role in directly eliminating reactive oxygen metabolites such as superoxide (O_2_^•^) and hydroxyl ions (OH^•^) and converting H_2_O_2_ into H_2_O and O_2_. Therefore, these enzymes prevent oxidative damage[49]. In the present study, SOD and CAT activities were decreased in DMH-induced CRC, as observed in previous studies, although the results were not statistically significant [9,34,38]. These results were expected since oxidative stress was reported to increase as CRC carcinogenesis occurs [49].

Intestinal microbiome disruption is considered one of the main risk factors leading to the initiation and progression of CRC [50]. The study of gut microbiota is particularly relevant in this animal model of CRC since there is a two-way interaction between DMH and the gut microbiota, mimicking CRC development in humans [51]. DMH may induce a change in the gut microbiota, and the gut microbiota can metabolize DMH. Remarkably, the gut microbiota is essential in the pro-carcinogenic activity of DMH since germ-free rats have a lower incidence rate of colon tumors than conventional mice (only 20% of germ-free mice had 1,2-dimethylhydrazine-induced colon tumors, while 93% of conventional mice developed multiple colon tumors) [52]. However, the human gut microbiota has its intrinsic duality. While it is an indispensable source of essential nutrients to the human body and participates in many physiological processes [53], the overgrowth of some microbial groups has been consistently linked to colorectal carcinogenesis [54].

We analyzed the impact of DMH on bacterial species richness and diversity, but we did not observe a significant effect. However, we observed changes in the microbiota-derived from DMH administration, such as increased Firmicutes and Euryarchaeota phyla in CRC groups. In addition, higher levels of Firmicutes, Clostridia, Clostridiales, Peptostreptococcaceae, and *Blautia* in CRC rats and higher levels of Prevotellaceae and *Prevotella* in CTRL rats were also observed. Our results agree with Zhu et al. [10], who also used DMH. Likewise, Kuugbee et al. [55] observed higher levels of the *Clostridium* genus in the DMH-induced CRC group vs. the control group. In our study, *Clostridium* cluster XVIII and *Clostridium sensu stricto* (cluster I) were also enriched in the CRC groups. A recent study in humans described that enteric Archaea (*Methanosphaera* genus) was enriched in CRC patients compared with control individuals [56], agreeing with our study.

In addition to the DMH effect, we also analyzed the age-related changes in the fecal microbiota of healthy rats, comparing CTRL1 vs. CTRL2 groups. The CTRL2 group (17 weeks older than those from CTRL1) showed a higher abundance of Proteobacteria. These results align with a previous cross-sectional study from newborn to centenarian individuals, which showed an elderly cluster with a significantly higher abundance of Proteobacteria than newborns [57].

Histopathological lesions in the colon, small intestine, and cecum showed higher inflammation in DMH-exposed rats than CTRL rats. This could have been triggered by an overgrowth of methanogenic Archaea methanogenic microorganisms (Methanobacteriaceae family and *Methanosphaera* genus), as previously reported for *Methanosphaera stadmanae*, which induced a strong inflammatory response in bowel diseases [58]. Conversely, the short-chain fatty acid-producing bacteria *Prevotella* has been linked to a protective role against gut inflammation. Hirano et al. [59] found significantly lower levels of the *Prevotella* genus in inflamed colonic tissue from patients with ulcerative colitis, a risk factor for CRC development, than in healthy individuals. The lower levels of this bacterial group in CRC vs. control rats could be associated with colon inflammation and histopathological findings. Moreover, depleted levels of *Lactobacillus* in CRC rats could be involved in the oxidative status of the animals since this genus *was* shown to be protective and significantly improve antioxidant status in colonic tissue from rats with ulcerative colitis [60], evidenced by the increased activity of the antioxidant enzymes CAT and SOD, which were altered in CRC groups. Therefore, the impact of DMH by increasing certain pro-inflammatory-related bacterial species and decreasing other species with anti-inflammatory and antioxidant effects could suggest that the gut microbiota, depending on the relative abundance of certain microbial groups, might act simultaneously in a protective or pro-carcinogenic manner.

## 5. Conclusions

Based on the results obtained with our protocol, we conclude that the animal model relied on the i.p. administration of the carcinogenic agent DMH at a dose of 40 mg/kg for seven consecutive weeks (once a week) allows following the development of preneoplastic lesions and the associated gut microbiota remodeling. In fact, the overgrowth of Archaea and the histological signs of colon inflammation highlights the contribution of inflammation-associated pathways to CRC development in this animal model. Thus, we have shown here that this model can be helpful to study the effect of therapies in the early phases of CRC by modulating, for instance, CRC-related gut adaptation and intestinal inflammation.

## Figures and Tables

**Table 1:** Small intestine, cecum, and colon + rectum relative weight in all groups. Data are presented as mean ± SD.

Group	Relative Weight (g g^−1^)		
	Small Intestine	Cecum	Colon + Rectum
CTRL1 (*n* = 6)	2.10 ± 0.40	0.42 ± 0.05	0.86 ± 0.37
CRC1 (*n* = 6)	3.54 ± 1.41 ^a,b^	1.04 ± 0.70	0.85 ± 0.12
CTRL2 (*n* = 6)	2.27 ± 0.48	0.45 ± 0.04	0.77 ± 0.05
CRC2 (*n* = 6)	3.48 ± 1.48 ^a,b^	1.30 ± 1.00 ^c,d^	1.01 ± 1.48

^a^ Statistically different from the CTRL1 group (*p* < 0.0001); ^b^ Statistically different from the CTRL2 group (*p* < 0.0001); ^c^ Statistically different from the CTRL1 group (*p* < 0.01); ^d^ Statistically different from the CTRL2 group (*p* < 0.01). The control (CTRL1 and CTRL2) groups were injected with EDTA–saline solutions, and the DMH-induced CRC groups (CRC1 and CRC2) were injected with DMH. Groups 1 and 2 were euthanized 11 and 17 weeks after the first administration, respectively.

**Table 2:** Small intestine, cecum, and colon + rectum relative weight in all groups. Data are presented as mean ± SD.

Group	Microhematocrit (%)
CTRL1 (*n* = 6)	50.2 ± 2.4
CRC1 (*n* = 6)	50.4 ± 1.8 ^a^
CTRL2 (*n* = 6)	48.8 ± 1.5
CRC2 (*n* = 6)	45.5 ± 2.6 ^b^

^a^ Statistically different from the CRC2 group (*p* < 0.01); ^b^ Statistically different from the CTRL1 group (*p* < 0.05). The control groups (CTRL1 and CTRL2) were injected with EDTA–saline solutions, and the DHM-induced CRC groups (CRC1 and CRC2) were injected with DMH. Groups 1 and 2 were euthanized 11 and 17 weeks after the first administration, respectively.

**Table 3:** Serum levels of albumin, total protein, cholesterol, glucose, ALT, IL-6, CRP, myostatin, and ghrelin. Data are presented as mean ± SD.

Serum Markers	CTRL1 (*n* = 6)	CRC1 (*n* = 6)	CTRL2 (*n* = 6)	CRC2 (*n* = 6)
Albumin (g/L)	18.72 ± 4.07	29.25 ± 6.40	21.25 ± 8.64	27.62 ± 5.44
Total protein (g/L)	34.45 ± 5.26	43.28 ± 9.90	35.48 ± 10.04	39.35 ± 12.18
Cholesterol (mg/dL)	124.97 ± 11.15	131.92 ± 10.62	146.68 ± 55.07	135.65 ± 34.27
Glucose (mg/dL)	252.15 ± 44.52	270.52 ± 76.91	267.68 ± 68.75	261.42 ± 67.45
ALT (U/L)	40.75 ± 7.72	44.70 ± 9.36	39.57 ± 9.93	40.10 ± 10.01
IL-6 (AU)	6.45 ± 2.39	5.66 ± 1.85 ^b^	13.36 ± 2.92 ^a^	8.25 ± 4.64
CRP (AU)	9.70 ± 2.27	7.48 ± 1.76	16.46 ± 4.35	12.28 ± 7.33
Myostatin (AU)	8.84 ± 3.78	7.47 ± 2.68	18.35 ± 4.82	11.87 ± 6.85
Ghrelin (AU)	9.80 ± 4.20	8.12 ± 2.00 ^c^	21.41 ± 5.61 ^a^	14.16 ± 6.92

^a^ Statistically different from the CTRL1 group (*p* < 0.05); ^b^ Statistically different from the CTRL2 group (*p* < 0.05); ^c^ Statistically different from the CTRL2 group (*p* < 0.01). Abbreviations, ALT: alanine aminotransferase; AU: arbitrary units; IL-6: interleukin-6; CRP: C-reactive protein. The control groups (CTRL1 and CTRL2) were injected with EDTA–saline solutions and the CRC groups (CRC1 and CRC2) were injected with DMH. Groups 1 and 2 were euthanized 11 and 17 weeks after the first administration, respectively.

**Table 4:** Degree of inflammation of small intestine and cecum. Data are presented as mean ± SD.

Group	Animal	Small Intestine	Cecum
CTRL1 (*n* = 6)	1	1	2
	2	1	1
	3	2	2
	4	2	2
	5	1	1
	6	1	2
	Mean ± SD	1.33 ± 0.47	1.67 ± 0.47
CRC 1 (*n* = 6)	1	2	1
	2	2	2
	3	2	2
	4	2	2
	5	2	2
	6	2	2
	Mean ± SD	2.00 ± 0.00 ^a^	1.83 ± 0.37
CTRL2 (*n* = 6)	1	2	2
	2	1	2
	3	1	2
	4	2	2
	5	1	2
	6	2	1
	Mean ± SD	1.50 ± 0.50	1.83 ± 0.37
CRC2 (*n* = 6)	1	2	2
	2	2	2
	3	2	2
	4	2	2
	5	2	2
	6	2	2
	Mean ± SD	2.00 ± 0.00 ^a^	2.00 ± 0.00

^a^ Statistically different from the CTRL1 group (*p* < 0.05). The control groups (CTRL1 and CTRL2) were injected with EDTA–saline solutions, and the CRC groups (CRC1 and CRC2) were injected with DMH. Groups 1 and 2 were euthanized 11 and 17 weeks after the first administration, respectively.

**Table 5:** Incidence of histopathological proliferative lesions observed in rats’ colon. The percentage represents the number of animals with injury per total number of animals in the group.

Group	Normal	Mild to Moderate Dysplasia	Severe Dysplasia	Adenoma
CTRL1 (*n* = 6)	6/6 (100%)	---	---	---
CRC1 (*n* = 6)	3/6 (50%)	3/6 (50%)	---	---
CTRL2 (*n* = 6)	6/6 (100%)	---	---	---
CRC2 (*n* = 6)	2/6 (33.3%)	2/6 (33.3%)	1/6 (16.7%)	1/6 (16.7%)

**Table 6:** Degree of inflammation of rats’ colon. Data are presented as mean ± SD.

Group	Animal	Severity	Thickness	Epithelial Damage	Extension
CTRL1 (*n* = 6)	1	1	1	0	1
	2	1	1	0	1
	3	1	1	1	2
	4	1	1	0	1
	5	1	1	1	2
	6	1	1	0	1
	Mean ± SD	1.00 ± 0.00	1.00 ± 0.00	0.33 ± 0.47	1.33 ± 0.47
CRC 1 (*n* = 6)	1	1	1	0	2
	2	2	1	1	2
	3	2	1	2	3
	4	2	2	1	2
	5	2	2	1	2
	6	2	2	2	2
	Mean ± SD	1.83 ± 0.37 ^a^	1.50 ± 0.50	1.17 ± 0.69 ^a^	2.17 ± 0.37 ^a^
CTRL2 (*n* = 6)	1	1	1	1	1
	2	1	1	1	1
	3	1	1	1	1
	4	1	1	1	2
	5	2	1	2	2
	6	2	1	2	2
	Mean ± SD	1.33 ± 0.47	1.00 ± 0.00	1.33 ± 0.47 ^b^	1.50 ± 0.50
CRC2 (*n* = 6)	1	2	2	2	3
	2	2	2	2	2
	3	1	1	1	2
	4	2	1	1	2
	5	2	2	3	2
	6	2	1	1	2
	Mean ± SD	1.83 ±0.37 ^a^	1.50 ± 0.50	1.67 ± 0.75 ^c^	2.17 ± 0.37 ^a^

^a,b,c^ Statistically different from the CTRL1 group (^a^ *p* < 0.05; ^b^ *p* < 0.01; ^c^ *p* < 0.0001). The control groups (CTRL1 and CTRL2) were injected with EDTA–saline solutions, and the CRC groups (CRC1 and CRC2) were injected with DMH. Groups 1 and 2 were euthanized 11 and 17 weeks after the first administration, respectively.

**Table 7:** Incidence of histopathological proliferative lesions observed in rats’ colon. The percentage represents the number of animals with injury per total number of animals in the group.

Group	SOD (U/min/mg of Protein)	CAT (H_2_O_2_/min/mg of Protein)
CTRL1 (*n* = 6)	19.42 ± 2.39	51.10 ± 12.65
CRC1 (*n* = 6)	16.12 ± 1.43	37.77 ± 16.61
CTRL2 (*n* = 6)	15.27 ± 3.73	40.50 ± 1.43
CRC2 (*n* = 6)	10.91 ± 2.11	34.94 ± 3.33

## Data Availability

Data is contained within the article or Supplementary Materials.

## Supplementary Materials

The following supporting information can be downloaded at: https://www.mdpi.com/article/10.3390/biomedicines10020409/s1, Table S1: Tests’ results carried out on animals from Charles River for viruses and bacteria. Supplementary Figure S1: Linear discriminant analysis (LDA) effect size (LefSe) showing significant differences in the fecal microbiota of (A) rat controls 1 (CTRL1) and 2 (CTRL2), and (B) DHMH-induced rat groups 1 (CRC1) and 2 (CRC2).
