# Supplementary material for: An Integrative Approach to Characterize the Early Phases of Dimethylhydrazine-Induced Colorectal Carcinogenesis in the Rat
Source: Biomedicines. 2022 Feb 9;10(2):409. doi: 10.3390/biomedicines10020409 (PMC8962270; doi:10.3390/biomedicines10020409)
Supplement: Supplementary file 1 [file biomedicines-10-00409-s001.zip › biomedicines-1552992-supplementary.pdf]

biomedicines

MDPI

Supplementary Materials

An integrative approach to characterize the early phases of di-methylhydrazine-induced colorectal carcinogenesis in the rat

Rita Silva-Reis[1], Catarina Castro Ribeiro[1], Mariana Gonçalves[1], Tiago Ferreira[1], Maria João Pires[1,2], Carlos E. Iglesias-Aguirre[3], Adrián Cortés-Martín[3], María V. Selma[3], Juan Carlos Espín[3]; Elisabete Nascimento-Gonçalves[1], Alexandra Moreira-Pais[4,5], Maria J. Neuparth[5], Francisco Peixoto[6], Eduardo Rosa[1], Adelina Gama[2,7], Rita Ferreira[4], Paula A. Oliveira[1,2], Ana I. Faustino-Rocha[1,8,9*]

Table S1. Tests' results carried out on animals from Charles River for viruses and bacteria.

	AGENTS	STATUS
VIRUSES	*Rinderpest virus*	Negative
	Ribgrass mosaic virus	Negative
	H1	Negative
	Kilham Rat Virus	Negative
	Sialodacryoadenitis virus	Negative
	Theiler's Murine Encephalomyelitis Virus	Negative
	Reovirus	Negative
	Pneumonia Virus of Mice	Negative
	Myeloblastosis associated virus	Negative
	Lymphocytic choriomeningitis virus	Negative
**BACTERIA**	*Hantavirus*	Negative
	B. bronchiseptcs	Negative
	C. kutscheri	Negative
	M. pulmonis	Negative
	P. pneumotropica	Negative
	P. multocida	Negative
	Salmonella spp.	Negative
	S. moniliformis	Negative
	B Strep. Sp.	Negative
	Strep. peumoniae	Negative
	H. hepaticus	Negative
	H. bilis	Negative
	Helicobacter sp.	Negative
	CAR bacillus	Negative

(A)

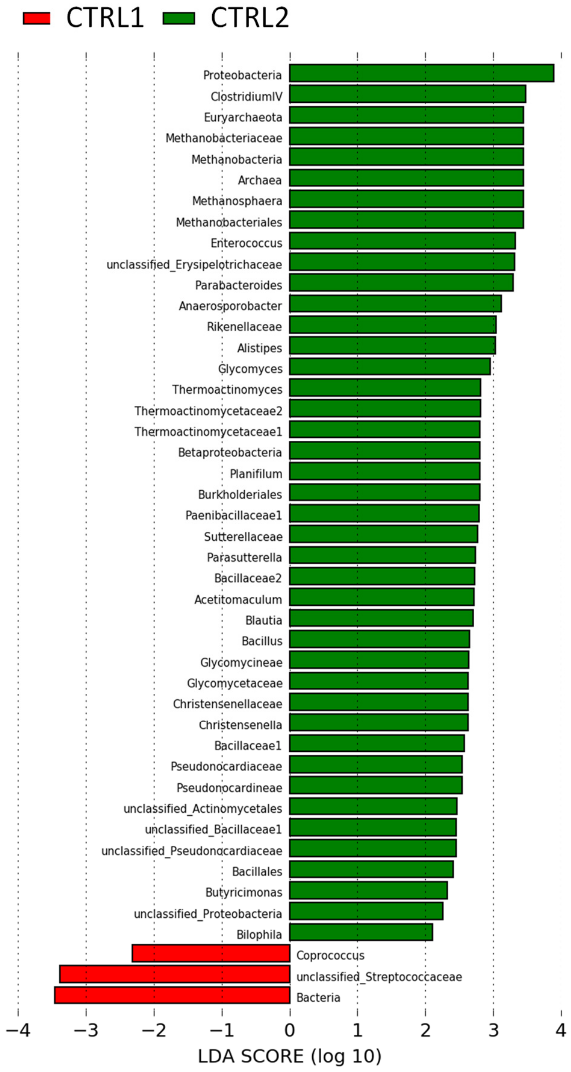
CTRL1
CTRL2
Proteobacteria
ClostridiumIV
Euryarchaeota
Methanobacteriaceae
Methanobacteria
Archaea
Methanosphaera
Methanobacteriales
Enterococcus
unclassified_Erysipelotrichaceae
Parabacteroides
Anaerosporobacter
Rikenellaceae
Alistipes
Glycomyces
Thermoactinomyces
Thermoactinomycetaceae2
Thermoactinomycetaceae1
Betaproteobacteria
Planifilum
Burkholderiales
Paenibacillaceae1
Sutterellaceae
Parasutterella
Bacillaceae2
Acetitomaculum
Blautia
Bacillus
Glycomycineae
Glycomycetaceae
Christensenellaceae
Christensenella
Bacillaceae1
Pseudonocardiaceae
Pseudonocardineae
unclassified_Actinomycetales
unclassified_Bacillaceae1
unclassified_Pseudonocardiaceae
Bacillales
Butyricimonas
unclassified_Proteobacteria
Bilophila
Coprococcus
unclassified_Streptococcaceae
Bacteria
−4 −3 −2 −1 0 1 2 3 4
LDA SCORE (log 10)

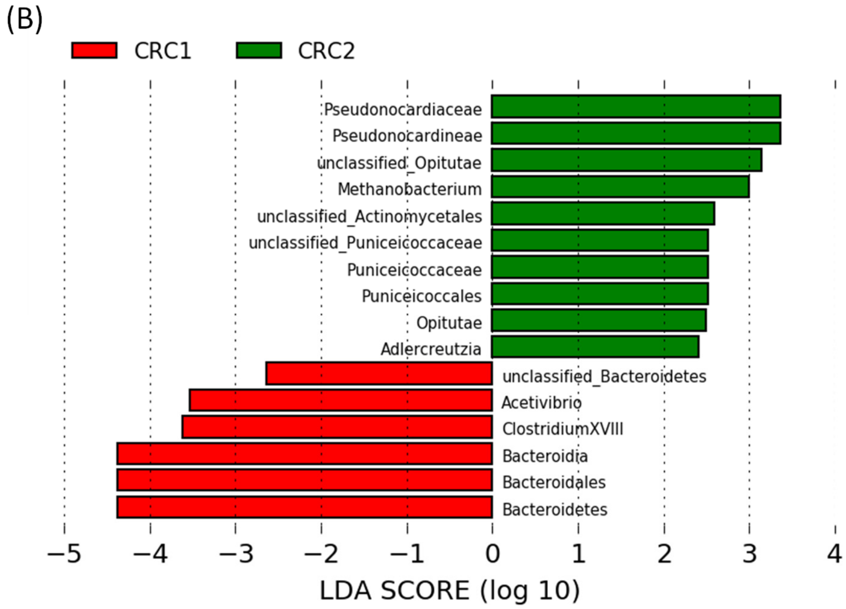

Supplementary Figure S1. Linear discriminant analysis (LDA) effect size (LEfSe) showing significant differences in the fecal microbiota of (A) rat controls 1 (CTRL1) and 2 (CTRL2), and (B) DHMH-induced rat groups 1 (CRC1) and 2 (CRC2).
